# L1 Hybridization Enrichment: A Method for Directly Accessing De Novo L1 Insertions in the Human Germline

**DOI:** 10.1002/humu.21533

**Published:** 2011-05-10

**Authors:** Peter Freeman, Catriona Macfarlane, Pamela Collier, Alec J Jeffreys, Richard M Badge

**Affiliations:** Department of Genetics, University of Leicester, University RoadLeicester, United Kingdom

**Keywords:** retrotransposon, human, LINE-1, L1, mutation rate, hybridization enrichment, germline, genome

## Abstract

Long interspersed nuclear element 1 (L1) retrotransposons are the only autonomously mobile human transposable elements. L1 retrotransposition has shaped our genome via insertional mutagenesis, sequence transduction, pseudogene formation, and ectopic recombination. However, L1 germline retrotransposition dynamics are poorly understood because de novo insertions occur very rarely: the frequency of disease-causing retrotransposon insertions suggests that one insertion event occurs in roughly 18–180 gametes. The method described here recovers full-length L1 insertions by using hybridization enrichment to capture L1 sequences from multiplex PCR-amplified DNA. Enrichment is achieved by hybridizing L1-specific biotinylated oligonucleotides to complementary molecules, followed by capture on streptavidin-coated paramagnetic beads. We show that multiplex, long-range PCR can amplify single molecules containing full-length L1 insertions for recovery by hybridization enrichment. We screened 600 µg of sperm DNA from one donor, but no bone fide de novo L1 insertions were found, suggesting a L1 retrotransposition frequency of <1 insertion in 400 haploid genomes. This lies below the lower bound of previous estimates, and indicates that L1 insertion, at least into the loci studied, is very rare in the male germline. It is a paradox that L1 replication is ongoing in the face of such apparently low activity. Hum Mutat 32:1–11, 2011. © 2011 Wiley-Liss, Inc.

## Introduction

Transposable elements are the most common class of repetitive DNA in the human genome, accounting for ∼45% of our DNA [Lander et al., 2001]. Short Interspersed Nuclear Elements (SINEs) account for 13% of the genome sequence, long interspersed nuclear elements (LINEs) for 20%, long terminal repeat (LTR) retrotransposons for 8%, and DNA transposons for 3% [Lander et al., 2001]. This accumulation of mobile DNA is apparently ongoing despite the fact that the most active known human transposable element, LINE 1 (L1), is relatively inactive compared to its counterpart in the mouse genome where ∼8% of spontaneous mutations arise through L1 retrotransposition [Ostertag and Kazazian, 2001]. This low activity is reflected in the rarity of L1-mediated pathogenic mutations identified in humans [Belancio et al., 2008; Kazazian and Moran, 1998; Xing et al., 2009].

With approximately 500,000 copies per human haploid genome [Lander et al., 2001] encompassing approximately 17% of human genomic DNA, L1 is the most prominent transposable element in humans, and in many other mammals [Lander et al., 2001; Moran and Gilbert, 2002]. However, 99.9% of these L1 copies are not able to retrotranspose [Moran et al., 1996] due to 5′ truncation or internal rearrangements [Boissinot et al., 2000; Moran and Gilbert, 2002]. There are around 90 full-length human L1s with intact open reading frames (ORFs) in the human genome reference sequence, which are therefore potentially retrotransposition-competent L1s (RC-L1s) [Brouha et al., 2003]. However, most RC-L1s are only weakly active in cell culture assays, with 6 of these 90 elements alone accounting for 84% of the total retrotransposition activity [Brouha et al., 2003]. It is not known whether this spectrum of activity is also seen in the germline.

L1 insertion into genes is known to have caused 17 cases of human genetic disease [Brouha et al., 2002; Divoky et al., 1996; Holmes et al., 1994; Kazazian et al., 1988; Kondo-Iida et al., 1999; Li et al., 2001a; Meischl et al., 1998, 2000; Miki et al., 1992; Mine et al., 2007; Morisada et al., 2010; Mukherjee et al., 2004; Narita et al., 1993; Schwahn et al., 1998; van den Hurk et al., 2003, 2007; Yoshida et al., 1998], accounting for approximately 1 in 1,200 human pathogenic mutations [Kazazian, 2004]. This incidence has allowed the frequency of L1 retrotransposition to be estimated variously as one in nine humans harboring a de novo L1 insertion somewhere in their genome [Kazazian, 1999], through 1 in 33 humans [Brouha et al., 2003] to as few as 1 in 186 humans [Li et al., 2001b]. Recently other estimates of L1 retrotransposition rates have been derived from comparisons between the L1 complement of the human genome reference sequence and entire individual diploid genome sequences [Xing et al., 2009] and through high-throughput L1-selective sequencing in 15 unrelated individuals [Ewing and Kazazian, 2010]. These sequencing-based estimates are at the lower end of previous analyses—1 in 212 live births [Xing et al., 2009]; 1 in 140 [Ewing and Kazazian, 2010]—despite being able to identify insertions within a significant proportion of the euchromatic genome.

Molecular parasites like L1 are often regarded as selfish DNA [Bestor, 1999; Hickey, 1982], under selection to maximize their copy number in following generations. For de novo L1 insertions to be of evolutionary consequence, they must occur in the germline or during embryogenesis prior to germline differentiation [Ergun et al., 2004]. Most disease-causing insertions are probably of germline origin as deleterious embryonic mutations are likely to be lost in development. Examples of germline pathogenic insertions are known: an insertion into the CYBB gene [Brouha et al., 2002] most likely occurred during prophase of maternal meiosis II, providing convincing evidence for retrotransposition in the female germline. Evidence for premeiotic insertions also exists, specifically in the case of an L1 insertion into the CHM gene, which must have occurred early in human female embryonic development because the transmitting individual is a somatic and germline mosaic [van den Hurk et al., 2003, 2007].

Direct analysis of L1 insertion in the female germline is prevented by the practical difficulty of obtaining oocytes. In contrast, sperm provide a readily-accessible resource for detecting de novo L1 insertions, provided that single DNA molecule methods can be developed to allow millions of sperm to be screened for insertions. Sperm analysis requires that L1 retrotransposition is ongoing in the male germline, and the evidence for this is circumstantial, but compelling. Immunohistochemical localization of L1 ORF1p, L1 ORF2p, and by inference L1 RNA, in adult and fetal human testes [Ergun et al., 2004] suggests that all the essential L1 retrotransposition components are present. Also, retrotransposition of tagged human L1 elements has been observed in spermatocytes of transgenic mice [Ostertag et al., 2002] and rats [Ostertag et al., 2007]. Finally, although there is no example of a disease-causing L1 insertion of unequivocally paternal origin, the existence of young polymorphic L1 insertions on the Y chromosome proves that L1 retrotransposition occurs in males [Santos et al., 2000].

De novo L1 insertions in the human germline have not been previously directly detected, except by chance in the case of disease–causing insertions, and so very little is known about the dynamics of L1 retrotransposition. Three factors have hampered attempts to access de novo L1 insertions. First, there are currently no human germline cell cultures. Second, L1 elements are relatively small insertions (1–6 kilobases [kb]) that can apparently insert anywhere within a large (3 Gigabase [Gb]) genome. Third, the frequency of insertion is likely to be extremely low, with the current estimates of de novo L1 activity predicting that a single insertion will occur in 1 in 9 to 1 in 186 humans, corresponding to a single de novo L1 insertion in 54 pg to 1.12 ng of germline DNA [Kazazian, 1999; Li et al., 2001b]. With such low frequencies, screening the whole human genome for de novo L1 insertions is currently not feasible.

Here we present the development of an L1 hybridization enrichment method capable of physically recovering complete L1 insertions into genomic targets devoid of L1 sequences. We illustrate the method's ability to recover full-length L1 insertions at the single DNA molecule level and present a study that enabled us to estimate an upper bound of the frequency of L1 retrotransposition in the human male germline at our selected loci.

## Materials and Methods

### Sperm DNA

Sperm DNA was prepared as described previously [Jeffreys et al., 1994] from semen samples collected with informed consent from two healthy volunteers (Donor A and Donor B) of north European origin, under ethical approval from the Leicestershire, Northamptonshire and Rutland Research Ethics Committee (LNRREC Ref. No. 6659 UHL).

### Target Locus Selection

**Figure 1:**
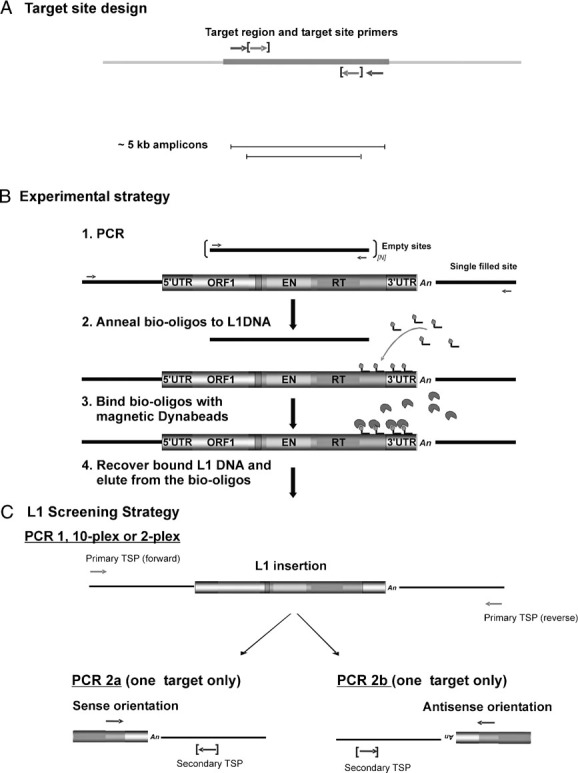
L1 hybridization enrichment strategy. **A:** Target site design. Schematic showing primary target site primers (TSPs, arrows) and secondary TSPs (bracketed arrows). The primary PCR amplifies a 5-kb empty target site. **B:** L1 amplification and hybridization enrichment. (1) A single filled site L1-containing molecule is present in a huge excess of empty site molecules. (2) Following primary PCR amplification, L1-containing amplicons are annealed to biotinylated L1-specific oligonucleotides (bio-oligos). (3) L1-containing amplicons are captured on streptavidin-coated paramagnetic beads. (4) L1-containing single-stranded DNA is released by thermal denaturation from the bead-bound bio-oligos. **C:** Screening enriched eluates for L1-containing targets. Full-length target molecules are amplified using primary TSPs (PCR1), then reamplified using appropriate combinations of an L1-specific primer together with a nested secondary TSP (bracketed) to target the L1/genomic DNA junction fragment, depending on the orientation of the insertion (PCR 2a or 2b). This nesting strategy prevents these amplicons becoming recoverable contaminants in subsequent MP-HE experiments.

Eight target loci known to have harbored disease-causing L1 insertions were selected for investigation, along with two additional loci (HoxD and MHC Class II) (Table 1). None of the genes associated with the target loci are known to have a role in spermatogenesis and so insertions in these genes are very unlikely to be selected against in sperm. Each target region sequence was screened for the absence of close matches (regions with three or fewer mismatches) to any of the biotinylated L1 specific oligonucleotides (L1 bio-oligos, detailed in Supp. Table S1). The sequences were also screened for the presence of multiple potential L1 integration sites [Yang et al., 1999], and using RepeatMasker open 3.0 (http://www.repeatmasker.org), to locate nonrepetitive DNA suitable for primary and secondary polymerase chain reaction (PCR) primer design, as shown in Figure 1A.

**Table 1:** Target Site Loci

Chr	Gene	Notes on locus	Reference	Amplicon length (*Bss*SI digested), bp^a^
X	DMD	X-linked dilated cardiomyopathy. Insertion into the 5′ UTR of the DMD gene	Yoshida et al. [1998]	4,909 (4,746 + 162)
X	F9	Heemophilia B. Insertion into exon 5 of the factor 9 gene	Li et al. [2001a]	5,552
X	CHM	Choroideremia (CHM). Insertion into exon 6 of the CHM gene	Van den Hurk et al. [2003]	5,678
X	CYBB	Chronic granulomatous disease (CGD). Insertion into intron 5 of the CYBB gene	Meischl et al. [2000]	5,160
X	RP2	Retinitis Pigmentosa 2 (RP2). Insertion into intron 1 of the RP2 gene	Schwahn et al. [1998]	4,774
11	HBB	B-Thalassaemia. Insertion into intron 2 of the Haemoglobin B gene	Kimberland et al. [1999]	5,006
9	FKTN	Fukuyama-type congenital muscular dystrophy (FCMD). Insertion into intron 7 of the FCMD gene	Kondo-Iida et al. [1999]	5,019
5	APC	Colon Cancer Susceptibility (APC). Potential disease causing insertion into exon 15 of the APC gene	Miki et al. [1992]	5,417
2	HOXD gene cluster	Homeo box D gene cluster. L1 repeat deficient	Greally [2002]	4,286
6	MHC2 region	MHC Class II region. Sequence variability well characterized	NA	5,018 (3,699 + 1,206 + 94 + 18)

a Length of the target amplicon, including fragment lengths following *Bss*SI digestion (in parentheses).

### Routine PCR

A total of 20 µl PCR reactions contained 20 to 50 ng of genomic DNA (gDNA), PCR buffer (45 mM Tris-HCl pH 8.8, 11 mM ammonium sulphate, 5 mM MgCl_2_, 6.7 mM 2-mercaptoethanol, 113 µg/ml BSA, 1.1 mM dNTPs), 0.05 µM of each primer and 0.025 U/µl 20:1 *Taq*/*Pfu* polymerases (Abgene, Epsom, UK; Stratagene, La Jolla, CA). PCR cycling was performed in an MJ Tetrad PTC250 thermal cycler (MJ Research/Biorad, Hercules, CA) at 96°C for 1 min, then at 96°C for 20 sec, 62°C for 1 min/kb of target amplicon plus 1 min for 30 cycles, followed by 61°C for 30 min. HPLC purified primers were supplied by Thermo Electron, and handled under PCR-clean conditions.

### Multiplex PCR

A total of 50 µl multiplex PCRs contained 500 ng gDNA, PCR buffer and *Taq*/*Pfu* as above. The concentrations of the primary target site primers in the multiplex PCR are shown in Supp. Table S2. PCR cycling conditions were: 96°C for 1 min, followed by 20 cycles of 96°C for 20 sec, 62°C for 14 min, and then 61°C for 30 min.

### Determining the Number of Amplifiable DNA Molecules

Genomic DNA samples were serially diluted in 10-fold steps using single molecule diluent (5 mM Tris-HCl pH 7.5, 5 µg/ml sonicated *Escherichia coli* genomic DNA) to an estimated concentration of 1 haploid genome/µl. PCRs were carried out in eight replicates of 1 µl input per dilution, then diluted 10-fold in 5 mM Tris HCl (pH 7.5) and 2 µl of the dilution used to seed nested secondary PCRs. Secondary PCR products were fractionated by agarose gel electrophoresis in the presence of 0.5 µg/ml ethidium bromide. The frequency of positive and negative reactions was used to Poisson estimate the maximum likelihood number of amplifiable molecules and its 95% confidence intervals [Jeffreys et al., 1994].

### PCR Product Purification

One-third of all primary PCR reactions from a single 96-well plate were pooled and purified by phenol/chloroform extraction using Phase Lock tubes (Eppendorf, Cambridge, UK) to remove oligonucleotide primers and DNA polymerase that could interfere with hybridization enrichment. The aqueous phase was reextracted with chloroform and the purified DNA collected by ethanol precipitation. DNA was redissolved in 33 µl 5 mM Tris-HCl (pH 7.5) prior to hybridization enrichment.

### Hybridization Enrichment

The principal stages of L1 hybridization enrichment are illustrated in Figure 1B, and described in detail below.

### Bead Preparation

M-280 streptavidin-coated super-paramagnetic Dynabeads (Invitrogen, Dynal, Paisley, UK) were captured with a Dynal MPC-S magnetic particle concentrator and washed three times at room temperature (resuspending each time) with 100 µl 1 × denaturing/hybridizing/binding buffer (DHB; 45 mM Tris-HCl pH 8.8, 11 mM ammonium sulphate, 4.5 mM MgCl_2_, 6.7 mM 2-mercaptoethanol, 4.4 µM EDTA, 2 µg/ml single-stranded (heat denatured) high molecular weight herring sperm DNA). The washed beads were resuspended in a volume of 1 × DHB 1/12th that of the original volume. This working stock of beads was kept in the dark, on ice.

### Annealing

Annealing (Fig. 1B, step 2) was carried out in 0.2 ml PCR tubes containing 33 µl of purified and concentrated multiplex PCR product, 4 µl 10 × DHB, 3 µl 5 µM biotinylated oligonucleotide (bio-oligo) mixture (0.375 µM final concentration) in a total volume of 40 µl. The mixture was denatured in a thermal cycler at 96°C for 75 sec followed by step-down annealing, in 1°C steps with 20 sec incubation at each step, from the optimal annealing temperature (A°) + 9°C to A° + 1°C. Annealing was completed by a final incubation at A° for 2 min. For the mixture of biotinylated L1 specific oligonucleotides used here (detailed in Supp. Table S1) A° was determined to be 38°C.

### Binding

Binding of the bio-oligo/DNA hybrids to Dynabeads (Fig. 1B, step 3) was carried out by transferring annealed DNA to a prewarmed siliconized eppendorf tube in a water bath at A°, then adding 3.6 µl of the working stock of Dynabeads and mixing, very gently, every 2 min for 10 min. Dynabeads were then captured on the magnetic particle concentrator, and the supernatant transferred to a fresh 0.2 ml PCR tube containing 3 µl of 5 µM bio-oligo mix, for reextraction (see below). The Dynabeads were washed gently in 100 µl of 1 × DHB + 10 µg/ml BSA on ice, transferred to a fresh siliconized 1.5 ml eppendorf tube on ice, captured on the concentrator and washed again with 100 µl prewarmed DHB + BSA at A° for 2.5 min. The Dynabeads were again captured and further washed at room temperature in 100 µl of Elution buffer (ED; 0.14 × DHB, 4.7 µg/ml single-stranded high molecular weight *E. coli* DNA) prior to transfer to a fresh siliconized Eppendorf tube. The Dynabeads were finally captured and resuspended in 50 µl ED prior to thermal elution.

### Recovery

Single-stranded DNA was recovered from bead-bound bio–oligos (Fig. 1B, step 4) through thermal elution, by placing the tubes in a 65°C water bath for 5 min. The Dynabeads were captured and the eluate, containing the released single-stranded DNA, was transferred to a 0.2 ml microcentrifuge tube on ice.

### Reextraction

The unbound fraction collected at the first cycle of enrichment was reextracted, by adding more bio-oligos and following the annealing/binding/recovery procedure as above, to maximize DNA recovery. In total, one extraction and two reextractions were carried out per sample. The eluates from the extraction and the reextractions were pooled. A total of 33 µl of each pooled eluate was then subjected to a second round of hybridization enrichment as above, and again eluates from the secondary extraction and the reextractions were pooled. All eluates and washes were stored in the dark at 4°C.

### Identification of Putative De Novo L1 Insertions

The eluted DNA was subjected to nested PCR amplification using secondary target site primers (Supp. Table S3) as shown in Figure 1C. Aliquots of PCR products were resolved by agarose gel electrophoresis, transferred to nylon membranes by Southern blotting, and hybridized with a ^32^P-labeled L1-specific oligonucleotide probe (PFLR5999). L1-sequence containing PCR products were detected by autoradiography. The remaining PCR products were fractionated by gel electrophoresis and stained with ethidium bromide (0.5 µg/ml). L1 sequence-containing bands identified by autoradiography were visualized using a Dark Reader trans-illuminator (Clare Chemical Research, Dolores, CO) and excised from the gel. DNA was extracted using the QIAquick gel extraction kit (Qiagen, Crawley, UK), cloned and sequenced (see below).

### Cloning and Sequencing PCR-Amplified DNA

Purified amplicons were ligated into the pGEM®-T Easy plasmid vector (pGEM®-T Easy Vector System I kit, Promega, Southampton, UK), following the manufacturer's protocol, and transformed into ultra competent DH5α *E. coli* cells. Plasmid DNA was recovered using the QIAprep Spin miniprep kit (Qiagen, Crawley, UK). A total of 20–30 ng/kb of plasmid DNA was sequenced using the Big Dye Terminator v3.1 ReadyReaction system (Applied Biosystems, Foster City, CA) with M13F or M13R sequencing primers. Excess reaction components were removed using PERFORMA DTR Gel Filtration Cartridges (Edge BioSystems Ltd, Gaithersburg, MD) and samples were analysed on an ABI3730XL capillary sequencer (Applied Biosystems).

### Analysis of Putative De Novo L1 Insertion Sequences

The entire sequence of cloned amplicons was assembled from sequence traces using the Align tool of the NCBI BLAST server Website (http://www.ncbi.nlm.nih.gov/blast/bl2seq/wblast2.cgi) and the GCG package (Accelrys Inc., San Diego, CA). Assembled sequences were aligned with the human L1 element L1.3 (accession L19088) and the appropriate target site sequence (Sequence Accessions are listed in Table 1) using the fasta algorithm in GCG. Putative de novo L1 insertions sequences showing regions of high identity to both the target site and L1.3 were exported from GCG and manually annotated.

## Results

### Strategy for Detecting De Novo Insertions

Even at the highest estimated frequency of L1 retrotransposition in the human germline (one in nine humans harboring a de novo L1 insertion somewhere within a 6 Gb diploid genome) [Kazazian, 1999], it would be necessary to screen 54 Gb sperm DNA to detect a single L1 insertion. This is not practical with current technology. Instead, we screened sperm for de novo insertions within selected genomic intervals devoid of L1 sequences. Because long PCR can efficiently amplify regions of 10 kb or more at the single DNA molecule level, we chose 5-kb long insertion targets; if such a target acquired a full-length 6 kb L1 insertion, then the resulting 11-kb DNA fragment would still be amplifiable and could be subsequently purified by hybridization enrichment using L1 specific probes (Fig. 1B). At the highest estimated frequency of retrotransposition, such de novo insertions into a single target should occur on average once per 10^7^ sperm. The efficiency of insert detection was further increased by amplifying ten different 5-kb targets prior to hybridization enrichment. This approach should in principle yield complete insertions suitable for structural analysis.

### Target Site Selection

Ten target loci (Table 1) were selected based on three criteria: amenability to L1 insertion, lack of L1 sequences, and suitability for efficient long PCR amplification (see Materials and Methods). Eight of these loci can accept L1 insertions because they have previously been the targets of disease-causing L1 insertions [Kimberland et al., 1999; Kondo-Iida et al., 1999; Li et al., 2001b; Meischl et al., 2000; Miki et al., 1992; Schwahn et al., 1998; van den Hurk et al., 2003; Yoshida et al., 1998]. We additionally selected the HoxD locus, a GC-rich target that is challenging for long PCR and unusually depleted in repetitive sequences [Greally, 2002], as well as an interval from the MHC class II region that was well characterized in the semen donor selected for the survey [Kauppi et al., 2003, 2004, 2005; Jeffreys et al., 2001]. Nested PCR primers were designed (Fig. 1A) that allowed each 5-kb target to be amplified efficiently at the single DNA molecule level. Together, these 10 targets would be expected to yield, at best, one de novo L1 insertion per ∼10^6^ sperm, or ∼100 insertions per ejaculate (∼10^8^ sperm).

### Multiplex PCR

**Figure 2:**
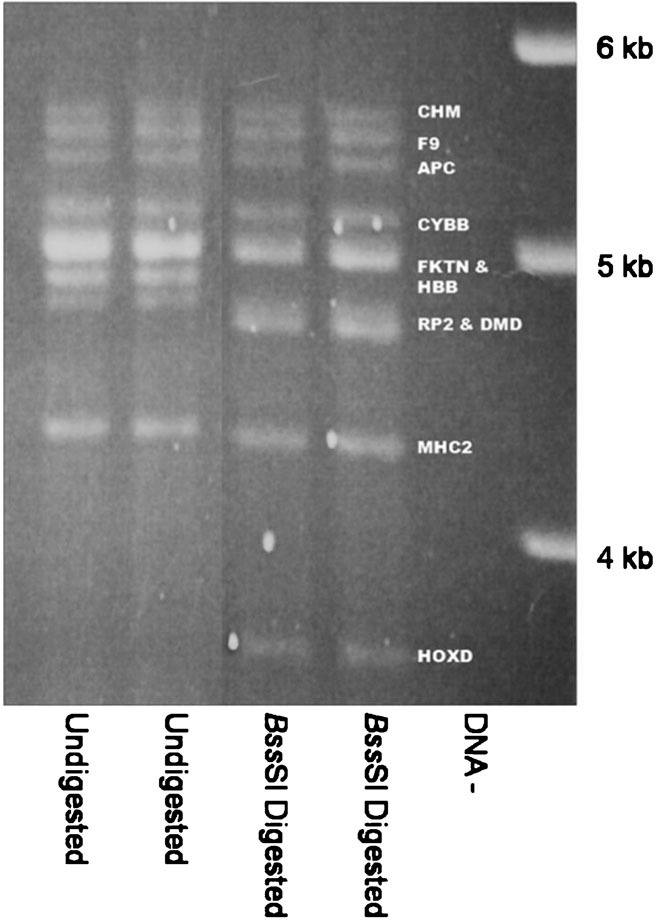
Multiplex PCR amplification of target loci. All 10 target loci were amplified from genomic DNA in a 10-plex PCR reaction. PCR products were analyzed by agarose gel electrophoresis, before or after digestion with *Bss*SI, as indicated. Amplicon sizes are shown in Table 1. DNA−, negative control reaction with no genomic DNA. Target identities in the *Bss*SI digest are shown using the identifiers in Table 1. Targets FKTN and HBB are not fully resolved but show approximately doubled band intensity, as expected for two comigrating fragments. This is also the case for the RP2 and DMD targets.

Thermal cycling conditions were optimized to ensure efficient amplification of all 10 targets in a single 50-µl multiplex PCR seeded with 0.5 µg of sperm DNA, the maximum DNA input compatible with efficient PCR. Digestion of the 10-plex secondary PCR products with *Bss*SI allowed identification of DNA fragments derived from each target (Table 1); these fragments were fairly uniform in intensity (Fig. 2), indicating that all targets were amplified with similar efficiency. The identity of each amplicon was confirmed by Southern blotting and hybridization with ^32^P-labeled target site-specific oligonucleotide probes (data not shown).

**Figure 3:**
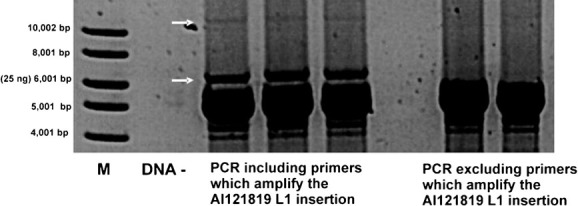
Amplification of a full-length L1 insertion in a multiplex PCR. Genomic DNA from donor A, heterozygous for the polymorphic AL121819 L1 insertion, was amplified using primers for all 10 target loci (10-plex, rightmost lanes) or for the 10 target loci plus the AL121819 insertion (11-plex, leftmost lanes). PCR products were analysed by agarose gel electrophoresis. DNA−, negative control; M, 1-kb DNA ladder (NEB). The 11-plex PCR shows two additional products (arrowed) corresponding to the empty AL121819 allele (6 kb) and the filled allele (12 kb).

To test whether multiplex PCR could also efficiently amplify molecules carrying a full-length L1 insertion, we added an extra primer pair specific for a locus containing the polymorphic AL121819 L1 insertion [Badge et al., 2003]. This 11-plex PCR generated two additional amplicons from an individual (donor A) showing presence/absence heterozygosity for this insertion: a 6-kb amplicon from the empty site and a 12-kb amplicon from the filled site (Fig. 3, white arrows). This demonstrated that the multiplex PCR could amplify full-length L1 insertions.

### Differential Amplification of Molecules Containing or Lacking L1 Inserts

Filled-site targets (12 kb) are much less efficiently amplified than empty sites (6 kb), as evident in Figure 3 when comparing the yield between upper and lower arrowed amplicons. To determine the extent to which this reduced efficiency is caused by the presence of damaged and unamplifiable molecules, we used nested PCR to amplify the control target containing the polymorphic AL121819 L1 insertion from limiting dilutions of donor A gDNA. We found that one amplifiable molecule carrying the insertion was present per 12 pg of gDNA (95% confidence interval [CI] 7–19 pg), while one amplifiable empty-site molecule was detected per 6 pg of gDNA (95% CI 4–9 pg). Given a diploid genome size of 6 pg, this suggests a single molecule PCR efficiency of ∼50% for filled sites and ∼100% for empty sites, and demonstrates that the low yield of filled-site PCR products (Fig. 3) is mainly due to inefficient amplification of long DNA molecules, rather than damaged template molecules.

To quantify the effect of this low PCR efficiency on de novo insert amplification and recovery, we analyzed the PCR product yield of an 11-plex PCR seeded with 0.5 µg of donor A gDNA, effectively containing ∼80,000 amplifiable molecules of the empty AL121819 site and ∼40,000 molecules of the filled site. This analysis indicated a gain of PCR products per cycle, from the empty and filled sites, respectively, of ∼1.8 and ∼1.6 over 20 cycles of PCR. This differential efficiency means that amplification of 0.5 µg gDNA containing a single amplifiable insertion molecule would produce 12,000 filled site molecules present in a huge excess of empty site molecules (10^11^ molecules over all 10 targets). It was therefore essential to recover de novo insertion PCR products by hybridization enrichment.

### Hybridization Recovery of L1 Insertions from PCR-Amplified Sperm DNA

DNA enrichment by allele–specific hybridization (DEASH) [Jeffreys and May, 2003] can be used to enrich specific DNA sequences, so we based L1 hybridization enrichment on a modified DEASH protocol (Fig. 1). Hybridization enrichment was performed using an equimolar mixture of four bio-oligos complementary to the most conserved sequences within the 3′ terminal 1.5 kb of young L1 subfamilies (Supp. Table S1). As L1 reverse transcription is initiated at the 3′ end of the element, restricting the bio-oligo sites to the 3′ terminus allows 5′ truncated insertions to be recovered.

Following two rounds of optimized hybridization enrichment, we routinely recovered at least 2% of PCR-amplified L1-containing molecules, compared with <4.5 × 10^−6^% of empty target site molecules. This indicates a >500,000 fold enrichment of L1-containing amplicons. After multiplex PCR, a pool of DNA would contain ∼12,000 L1-containing molecules derived from each de novo insertion, plus ∼10^11^ empty target site molecules. Following a single round of enrichment, this ratio of filled to empty site molecules would increase from 1/8,000,000 to >1/16, allowing a single molecule of a de novo insertion to be readily detected.

### Amplification and Recovery of an L1-Containing Target at the Single Molecule Level

**Figure 4:**
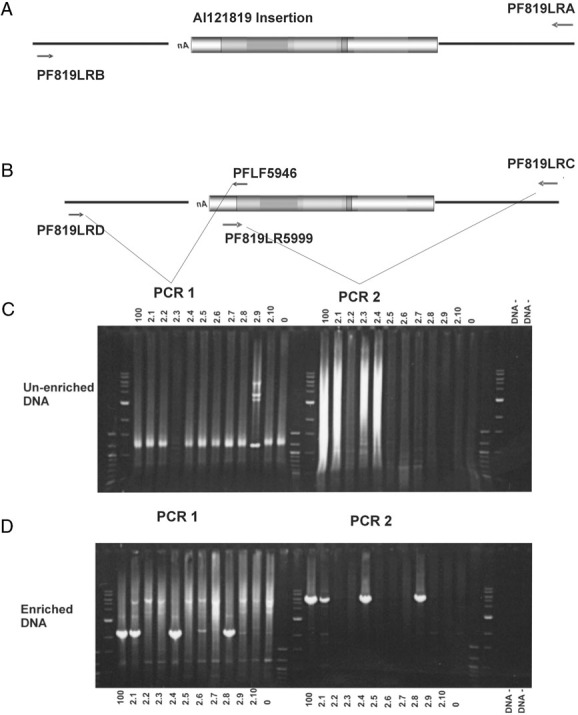
Hybridization-enrichment recovery of L1 insertions at the single molecule level. Results of a DNA mixing experiment in which pg amounts of gDNA from a heterozygous carrier of the L1 insertion in accession AL121819 were mixed with 48 µg of gDNA from an individual lacking the insertion (**A, B**). Multiplex PCR was performed on the DNA mixtures and the amplicons were then either not enriched (**C**) or subjected to hybridization enrichment (**D**). A: Enriched and unenriched amplicons were seeded into primary PCRs selective for the AL121819 locus, amplifying both filled (L1 insertion present) and empty (L1 insertion absent) DNA. B: Primary PCR products were subjected to two different secondary PCRs: PCR 1 selectively amplifies the 3′ end of the insertion, and PCR 2 selectively amplifies the 5′ end of the insertion. C: Without hybridization enrichment no L1 specific amplicons are obtained. Lanes labeled “100” contain secondary PCR products derived from DNA mixtures containing ∼100 molecules of L1 insertion containing gDNA, in 48 µg of insertion lacking gDNA. Lanes labeled “2.1” through “2.10” are DNA mixtures each containing gDNA with ∼2 molecules of L1 insertion, in 48 µg of insertion-lacking gDNA. Lanes labeled 0 contain only insertion-lacking gDNA. PCRs were fractionated alongside 250 ng 100 bp DNA ladder and 250 ng 1 kb DNA ladder (NEB), respectively. gDNA-free negative control reactions are labelled “DNA−.” D: When hybridization enrichment was performed, L1-specific PCR products were produced, with precise concordance between the PCR 1 and PCR 2 results indicating that entire insertions had been recovered. Lanes are labeled as in C.

To test whether DEASH could recover full-length L1 insertions at the single DNA molecule level, we mixed 24 pg sperm gDNA from donor A containing ∼2 amplifiable molecules of the AL121819 insertion (see above) with 0.5 µg of sperm gDNA from donor B, who lacks the AL121819 insertion. This DNA mixture, along with 95 additional reactions each containing 0.5 µg of sperm gDNA from donor B alone, was subjected to 11-plex PCR to amplify all ten targets plus the AL121819 locus. Amplified DNA from all 96 reactions was pooled and purified, and one-third of this DNA subjected to L1 hybridization enrichment. PCR amplification of the AL121819 target, either alone or as part of an 11-plex PCR for all targets, was followed by reamplification using primers designed to separately amplify the 5′ and 3′ junctions of the AL121819 insert (Fig. 4A). These PCRs generated appropriately sized junction fragment products (Fig. 4B, lower panel), whose identity was confirmed by locus specific and L1 specific oligonucleotide hybridization (data not shown). These products were not detected by PCR amplification of the unenriched DNA (Fig. 4B, upper panel). This model experiment established that L1 insertion molecules could be amplified from a huge excess (2 × 10^6^ fold) of insert-free genomic DNA, and that hybridization enrichment was essential for their detection.

### Screening Human Sperm DNA for De Novo L1 Insertions

Having established that hybridization enrichment could recover insertions at the single DNA molecule level, we screened 576 µg sperm gDNA from donor B for de novo L1 insertions. DNA was amplified by 10-plex PCR in 0.5 µg aliquots distributed over 12 96-well plates, and the PCR products from each plate pooled and enriched as above. Each of the 12 batches of enriched DNA was then screened for L1–containing target molecules using 10-plex (or duplex) primary PCR followed by nested PCRs as shown in Figure 1C. Eleven putative L1 insertion amplicons were identified. Their structures are summarized in Supp. Figure S1.

**Figure 5:**
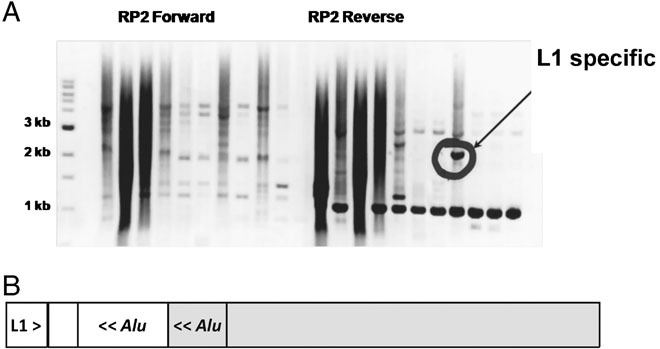
Structure of L1 insertion artifacts. **A:** Fractionation of amplicons from the RP2 target by agarose gel electrophoresis. Multiple PCR products were observed in each lane. Only one product (circled) was positive by L1-specific Southern blot hybridization. **B:** Structure of the L1-positive DNA fragment as established by DNA sequencing. The amplicon consisted of the 3′ end of a human specific L1 element and its flanking sequences mapped to chromosome 17 (white boxes), fused to the RP2 target on chromosome X (gray boxes). The fusion junction most likely occurred in the A-rich linker region found between the monomers of an AluSx element (gray box) in the RP2 target and an AluSg element (white box) at the chromosome 17 locus, thus forming an intact chimaeric Alu element. The 5′–3′ orientation of the repeat sequences is indicated by <<and > symbols. PCR of the enriched DNA failed to yield the expected amplicon corresponding to the 5′ end of the recovered L1 fused to the RP2 target sequence.

A genuine de novo L1 insertion into one of the target loci should contain an L1 sequence and a poly A tail, flanked 5′ and 3′ by target site sequences. All of the 11 putative insertion amplicons showed sequence similarity to one of the target loci (for an example, see Fig. 5B). However, most also contained sequences unrelated to the target site (Fig. 5B). Additional diagnostic PCRs, designed to amplify the inferred 5′ junction of L1 insertion amplicons, failed to identify any such junction (data not shown). This strongly suggested none of the recovered sequences had the structure predicted for a complete de novo L1 insertion and were therefore most likely not genuine insertions.

## Discussion

Our understanding of L1 insertion in the human germline currently rests entirely on L1 insertion/deletion polymorphisms in populations and on the chance observation of rare pathogenic de novo insertions. We aimed to develop a method for directly detecting de novo L1 insertion events in genomic DNA. L1 elements can insert anywhere into the genome [Feng et al., 1996; Moran, 1999] and L1 display methods could in principle be used to scan sperm DNA for such de novo insertions [Badge et al., 2003]. In practice, this approach is limited by the very low L1 insertion frequency, by incomplete genome coverage, and by its ability to recover only short L1/genomic DNA junctions yielding only partial information on insertion structure and with no guarantee that such junctions are not PCR artefacts. Recent approaches using L1 specific PCR amplification combined with High Throughput (HT) sequencing [Ewing and Kazazian, 2010; Iskow et al., 2010] or microarray hybridization [Huang et al., 2010] can in principle detect de novo L1 insertion/genomic junctions genome-wide. However, in the case of single molecule insertions whose originating DNA fragments are unavoidably destroyed during PCR amplification, these approaches can again only yield unverifiable partial junction sequences of low information content (HT sequencing) or simple presence/absence data (microarray hybridization). Finally, while fosmid library-based end sequencing approaches can capture intact L1 insertions [Beck et al., 2010], current estimates of retrotransposition frequency would require sequencing of >3 × 10^7^ fosmids, which would be prohibitively expensive and likely to generate false positives through rearrangement and chimaerism. In contrast, the present approach was designed to recover intact de novo L1 insertions that could be completely characterized by sequencing. The 10 targets selected only cover 0.0017% of the human genome, but this limitation is more than compensated for by the ability to screen huge numbers of sperm. Previous unsuccessful attempts to recover de novo insertions used physical selection of target amplicons based on an increase in DNA fragment size following insertion [Hollies et al., 2001]. In contrast, we used hybridization enrichment [Jeffreys and May, 2003], which provides far greater levels of purification and readily scales to very large inputs of genomic DNA. Our model experiment showed that single DNA molecules carrying a full-length insertion into a 6-kb target can be recovered by Multiplex PCR of target amplicons followed by Hybridization Enrichment (MP-HE), even in the presence of a huge excess of genomic DNA lacking the insertion.

We used MP-HE to survey 576 µg of sperm DNA from a single donor for de novo L1 insertions. Eleven putative L1 insertions were identified, all containing L1_HS_ or L1PA2 sequences, and all carrying at least one site fully complementary to the bio-oligos used for enrichment. However, none had a structure compatible with a canonical L1 insertion, excluding retrotransposition as an explanation for their origin. Instead, these molecules appear to be chimaeras between the target loci and known L1 insertions, most likely generated by strand jumping or template switching between sequences showing sequence similarity during the initial multiplex PCR. This is especially likely as the junction between the target site and the breakdown of similarity from the target site sequence was, in all cases an A/T rich tract (11/11) most often associated with Alu elements (10/11). In 7 of the 11 cases the L1 and flanking sequence were >99% identical to regions of the genome harboring known L1 elements. Although no genuine insertions were identified, these chimaeric artifacts do provide further validation of the MP-HE approach, showing that L1 hybrid molecules generated during PCR amplification can be recovered by our strategy, but are easily identified as artifacts by sequencing.

This major survey of sperm DNA from a single donor failed to yield any genuine insertions. These data can be used to estimate an upper bound of the frequency of L1 insertion in this man's germline, with the caveat that this estimate only applies to the selected target loci. Indeed, because most of the target loci have accommodated pathogenic insertions in the past we may have ascertainment bias in favor of insertion-prone loci. This bias is likely to cause overestimation of the insertion rate, making even more significant the lack of insertions detected here. The DNA analysed was derived from 1.9 × 10^8^ sperm, or 9.6 × 10^7^ amplifiable molecules of each target under the assumption that single molecule PCR is 50% efficient when amplifying a 12-kb amplicon, as established for control target molecules carrying full-length L1 insertions. The 10 loci surveyed together cover 51 kb of target DNA per sperm, within which a de novo insertion could be detected. As half of the loci are on the X chromosome, and so only present in 50% of sperm, this is effectively reduced to 38 kb of DNA per sperm. We have therefore screened 38 kb × 9.6 × 10^7^ = 3.7 × 10^9^ kb genomic DNA for insertions. The lack of insertions places an upper bound on the L1 insertion frequency of three insertions in 3.7 × 10^9^ kb (*P* = 0.05), or <1 event per 400 haploid genomes, lower than estimates of L1 retrotransposition frequency derived from the incidence of pathogenic L1 insertions in humans (range; 1/18–1/186) [Brouha et al., 2003; Kazazian, 1999; Li et al., 2001b] and from population diversity in genomic L1 complement (95% CIs 1 in 156–289) [Xing et al., 2009] and 1 in 95–270, [Ewing and Kazazian, 2010].

The reason for this very low estimated frequency of de novo insertion of L1 elements in an individual male germline is unclear. It is unlikely that the chosen target loci are refractory to insertion because L1 insertion into the genome appears to be largely random [Feng et al., 1996; Moran, 1999]. Also, 8 of the 10 targets were selected because they had accommodated known pathogenic insertions. These targets were also biased in favor of X-linked loci, reflecting the biased ascertainment of X-linked disease-causing L1 insertions exposed by hemizygosity in males. Also, the human X chromosome is nearly twofold enriched for L1 sequences compared to autosomes [Bailey et al., 2000; Lander et al., 2001; Ross et al., 2005], suggesting that it might either be a preferred target, or that X-linked L1 insertions are more likely to be fixed in the population. On balance, it therefore appears that the selected target loci are good proxies for the genome at large, although we cannot formally exclude the possibility that our target loci are refractory to insertion in this particular donor.

It is possible that donor B has an unusually low frequency of germline L1 retrotransposition due to the absence of active L1s in his genome. He does indeed lack the most active L1 identified to date, AC002980 [Brouha et al., 2003], but there are five remaining “hot” L1s that account for 63% of the summed activity in cell culture-based retrotransposition assays across all intact L1s identified in the human genome sequence [Brouha et al., 2003]. Given their allele frequencies [Brouha et al., 2003], it is likely that donor B carries at least one of these elements. Also, a recent genome-wide survey of full-length L1 elements showed that six individuals each harbored three to nine novel elements, of which 54% are active [Beck et al., 2010]. These numerous, rare active L1s in human genomes make it unlikely that the donor was substantially depleted for active L1s, although we cannot formally exclude this possibility without sequencing his genome and determining the activity of the RC-L1 elements that he carries.

Other factors could contribute to the very low rate of L1 retrotransposition observed here. Previously it was thought that L1 was primarily active during meiosis [Brouha et al., 2002], in which case specific germline L1 insertions should be nonrecurrent and occur at similar frequencies in different men harboring similar complements of active L1s. However, there is growing evidence that L1 mobilization can occur premeiotically, reflecting the existence of systems that actively repress L1 mobilization in meiosis [Bestor, 1999; Hata and Sakaki, 1997; Kierszenbaum, 2002; Li, 2002; Mann, 2001; Walsh et al., 1998]. The most potent form of L1 repression operates by transcriptional silencing through promoter hypermethylation [Hata and Sakaki, 1997; Schulz et al., 2006; Walsh et al., 1998]. Removal of these blocks by genomic hypomethylation occurs at two stages of early embryogenesis [Brandeis et al., 1993], providing two potential windows of opportunity for the expression of mobile elements and thus retrotransposition [Brandeis et al., 1993; Georgiou et al., 2009]. If L1 elements insert at the blastocyst stage, before germline partitioning, this could generate high level somatic and germline mosaicism, with many cells sharing the same insertion and resulting in high-frequency transmission of insertions to the next generation. Such “jackpot” de novo L1 insertions are supported by recent experimental data [Garcia-Perez et al., 2007; van den Hurk et al., 2007]. It is therefore possible that a small proportion of individuals in the human population could have a high L1 insertion load through mosaicism, and thus contribute most new insertions to the next generation. This raises the question of variation between individuals in the frequency of retrotransposition and whether some men show a high frequency of de novo insertions, with multiple copies of the same insertion signalling mosaicism. Our MP-HE method is suitable for such a survey, although its feasibility will depend on the frequency of men showing detectable mosaicism, and as such is outside of the scope of the pilot experiments presented here. Unfortunately current population-averaged estimates of transposition frequency reflect the combined effects of postinsertion selection, the frequency of mosaic individuals, and the levels of mosaicism within them, but give us no clues about the likely prevalence of such mosaic men.
