# Supplementary material for: L1 Hybridization Enrichment: A Method for Directly Accessing De Novo L1 Insertions in the Human Germline
Source: Hum Mutat. 2011 May 10;32(8):978–88. doi: 10.1002/humu.21533 (PMC3412225; doi:10.1002/humu.21533)
Supplement: Supplementary file 1 [file humu0032-0978-SD1.pdf]

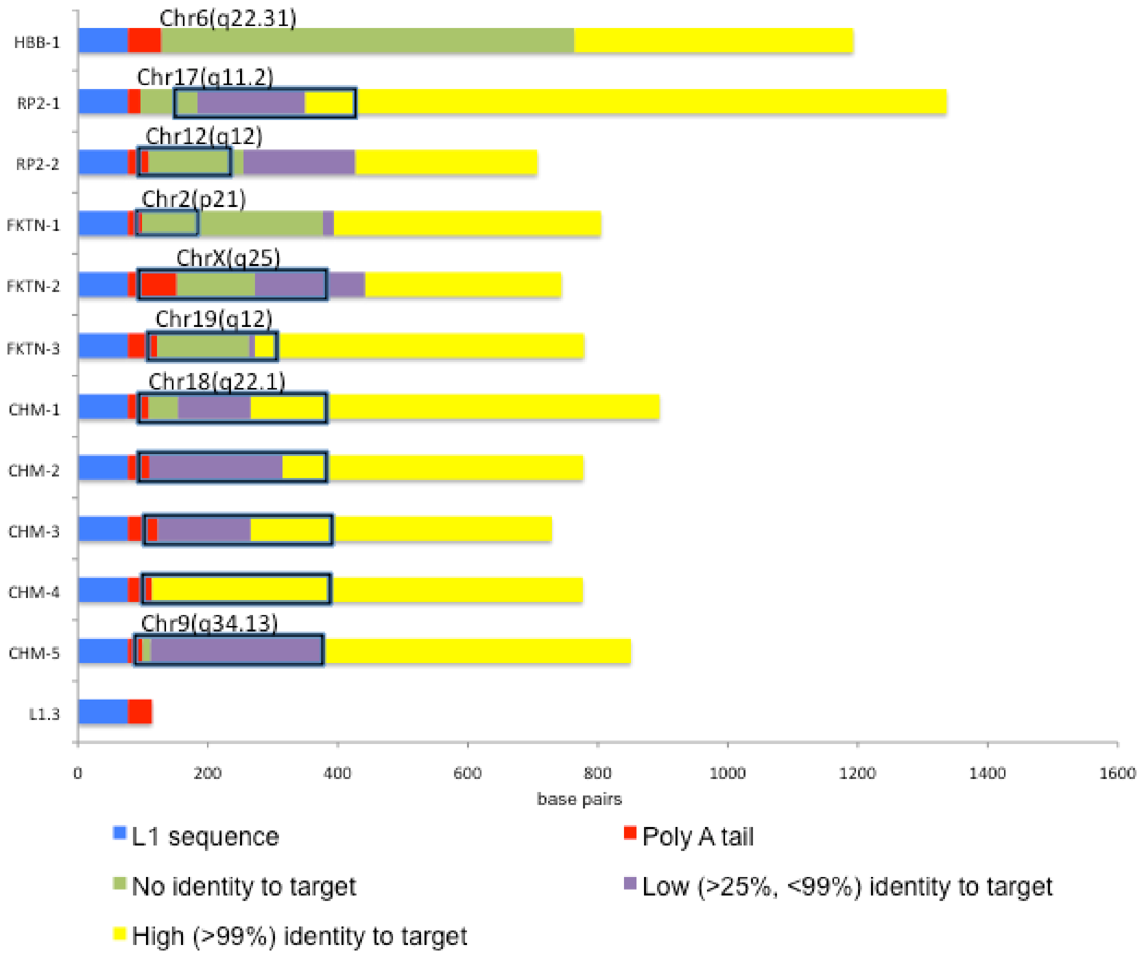

Supp. Figure S1. Structures of the 3′ ends of 11 potential *de novo* L1 insertions recovered by hybridisation enrichment, aligned to the human-specific L1 insertion, L1.3 (L19088). The names of the sequences include the target locus from which they originate. Bars are coloured to show the origin of the sequence: the L1 sequence is blue, the L1 poly A tail is red, regions of no alignable identity to the target locus are green (with chromosome origin shown above, where identified), regions of intermediate identity to the target sequence (>25% to <99%) are purple, and regions of high (>99%) identity to the target sequence are yellow. Regions containing Alu sequences likely involved in template switching are boxed and the size of the boxes reflects the length of the Alu tract.

Supp. Table S1. Biotinylated oligonucleotides used for hybridisation enrichment

**Bio-oligo ID**	**Sequence (5′- 3′)**	Position (L1.3[1])
bio-L1U1	bio-GGCACATGTATACATATG	5956
bio-L1U2	bio-GAAATACCATTTGACCCA	5489
bio-L1U3	bio-TGACAAAGGGCTAATATC	5124
bio-L1U4	bio-ACTACCTGACTTCAAACT	4602

[1] The location of each bio-oligo with respect to L1.3 (accession L19088)

Supp. Table S2. Primary target site PCR primers used for multiplex PCR and their optimised concentrations

Target Locus	Primer ID	Sequence	Working concentration (µM)	Accession Number	Location[1]	Size, bp[2]
	PFHBBTF1	ATTTGCCTGGTATGCCTGGG	0.07	ac104389	175485	
HBB	PFHBBTR1	CCTTGAAGCCAGGATGATGG	0.07	ac104389	180490	5005
	PFDMDTF1	GTGCTTTAGACATTACCCAGG	0.12	al031643	111421	
DMD	PFDMDTR1	CAACACATCTCTTCATACAGAGGTG	0.12	al031643	116329	4908
	PFFIXTF1	CACTGAGACCCCCTTCGG	0.08	al033433	17106	
F9	PFFIXTR1	CTGGTATAGTGCTGAGACAGG	0.08	al033433	22657	5551
	PFMH2TF1	TGCAGGGGCAGAAGAGGG	0.15	al662845	32532	
MHC2	PFMH2TR1	CCCCCTTGTCTTGCTAAGGG	0.15	al662845	37549	5017
	PFCHMTF1	TGAATGCTGGTTGTGGGAGG	0.05	al009175	57913	
CHM	PFCHMTR1	ACCATCTATGGTGCTGCTGG	0.05	al009175	63590	5677
	PFAPCTF1	GGAAGCATTATGGGACATGG	0.025	ac008575	127059	
APC	PFAPCTR1	CCTGAACAGACGAATGTGTGG	0.025	ac008575	132475	5416
	PFCGDTF1	GTTGGCTAACCATCAGAGGG	0.07	al627245	38073	
CYBB	PFCGDTR1	CAGCAAACTGAGGGATTGGG	0.07	al627245	43232	5159
	PFRP2TF1	CACAGAAGAGGATTGGGAGG	0.03	al050307	86064	
RP2	PFRP2TR1	TCTGTCATGCCCAACCTCTG	0.03	al050307	90837	4773
	PFFCMTF1	CCTTTCGGAAGAGTGCAAAGG	0.1	al158070	94766	
FKTN	PFFCMTR1	TGGAGCTAAGGTTTCCCAGG	0.1	al158070	99844	5078
	PFHODTF1	TTCGGTTCCTTCTCTGCTGG	0.1	ac009336	84862	
HOXD	PFHODTR1	CTCCTAAAATGGCTCCCTGG	0.1	ac009336	89126	4264
	PFLRCtrlA	TTAGGACGCCCACTACTGTG	0.1	ac008706	73112	
Control	PFLRCtrlB	GCCAATCCTGTAAGGCAAAGGG	0.1	ac008706	85917	12805
	PF819LRA	CCTGCTTTCACTTCACAGGG	0.05	al121819	105131	
Al121819 L1 insertion	PF819LRB	CAGGTAGAAACTTCCCAGGG	0.05	al121819	111585	6454

[1] Location of the primer within the sequence accession; [2] Length of the primary PCR amplicon.

Supp. Table S3. PCR primers used for secondary TSP multiplex PCRs

Target Locus	Primer ID	Sequence	Concentration (μM)	Accession Number	Location[1]	Size, bp[2]
	PFHBBTF2	CCTTGAAGCCAGGATGATGG	0.05	ac104389	175627	
HBB	PFHBBTR2	AGCCAGAAGCACCATAAGGG	0.05	ac104389	180351	4724
	PFDMDTF2	GTACCTCACAGCATAGAGGG	0.05	al031643	111738	
DMD	PFDMDTR2	GTTCTGTGTTGTCAGACAGGG	0.05	al031643	116167	4429
	PFFIXTF2	TCTATGGAACTCTCCCCTGG	0.05	al033433	17217	
F9	PFFIXTR2	GCCATACGAACATGGAGTGG	0.05	al033433	22443	5226
	PFMH2TF2	TAGCAACTGACTCCATGAGG	0.05	al662845	32604	
MHC2	PFMH2TR2	GAAACCTGGATAGAGACGTGG	0.05	al662845	37418	4814
	PFCHMTF2	ACCCTGAAGGAGACTTCTGG	0.05	al009175	57972	
CHM	PFCHMTR2	CCTCACAAAGGACATAGGTGG	0.05	al009175	63376	5404
	PFAP2TR2	GACCTAGTGGGAGAAGCTGG	0.05	ac008575	127170	
APC	PFAPCTF2	TAGGCCTGCGAAGTACAAGG	0.05	ac008575	132404	5234
	PFCGDTF2	TCCTCACCTCATGTGTGGAG	0.05	al627245	38145	
CYBB	PFCGDTR2	CACGTACAATTCGTCTGGGTG	0.05	al627245	43130	4985
	PFRP2TF2	GGTACAATTCTTGAGGGGGTG	0.05	al050307	86090	
RP2	PFRP2TR2	TCTGTCATGCCCAACCTCTG	0.05	al050307	90787	4697
	PFFCMTF2	TGGCTGAGCAGTGGAACTTG	0.05	al158070	94837	
FKTN	PFFCMTR2	TTGGACTGACAAACCCAGGG	0.05	al158070	99737	4900
	PFHODTF2	CCTTAAGGCTTCCACGTTGG	0.05	ac009336	84995	
HOXD	PFHODTR2	CGGGTCTTTATGTGTCTGGG	0.05	ac009336	89047	4052
Al121819 flanking primers	PF819LRC	TGTTCTCACAGCCTGACAGG	0.05	al121819	105208	
	PF819LRD	CTGGCTTGTAGGTACCAAGG	0.05	al121819	111536	6328

[1] Location of primer within the sequence accession; [2] Length of secondary PCR amplicon in base pairs.
